# The Potential of Genome-Wide Prediction to Support Parental Selection, Evaluated with Data from a Commercial Barley Breeding Program

**DOI:** 10.3390/plants11192564

**Published:** 2022-09-29

**Authors:** Maximilian Rembe, Yusheng Zhao, Neele Wendler, Klaus Oldach, Viktor Korzun, Jochen C. Reif

**Affiliations:** 1 Department of Breeding Research, Leibniz Institute of Plant Genetics and Crop Plant Research (IPK), D-06466 Gatersleben, Germany; 2 KWS LOCHOW GmbH, Ferdinand-von-Lochow-Str. 5, 29303 Bergen, Germany; 3 KWS SAAT SE & Co. KGaA, Grimsehlstr. 31, 37574 Einbeck, Germany

**Keywords:** usefulness criterion, variance prediction, long-term genomic selection, superior progeny

## Abstract

Parental selection is at the beginning and contributes significantly to the success of any breeding work. The value of a cross is reflected in the potential of its progeny population. Breeders invest substantial resources in evaluating progeny to select the best performing genotypes as candidates for variety development. Several proposals have been made to use genomics to support parental selection. These have mostly been evaluated using theoretical considerations or simulation studies. However, evaluations using experimental data have rarely been conducted. In this study, we tested the potential of genomic prediction for predicting the progeny mean, variance, and usefulness criterion using data from an applied breeding population for winter barley. For three traits with genetic architectures at varying levels of complexity, ear emergence, plant height, and grain yield, progeny mean, variance, and usefulness criterion were predicted and validated in scenarios resembling situations in which the described tools shall be used in plant breeding. While the population mean could be predicted with moderate to high prediction abilities amounting to 0.64, 0.21, and 0.39 in ear emergence, plant height, and grain yield, respectively, the prediction of family variance appeared difficult, as reflected in low prediction abilities of 0.41, 0.11, and 0.14, for ear emergence, plant height, and grain yield, respectively. We have shown that identifying superior crosses remains a challenging task and suggest that the success of predicting the usefulness criterion depends strongly on the complexity of the underlying trait.

## 1. Introduction

An important step in breeding is the selection of promising parents to initiate a breeding cycle [1]. Ideally, genetically complementary parents are crossed to initiate a breeding population from which the best performing candidates are selected to bring new varieties to market. The central question of optimal parental selection for breeding programs is probably as old as breeding itself. Despite an increasing number of theoretical considerations on strategies for selecting parents, few of them have found their way into practice, and many breeders rather rely on basic concepts such as crossing best times best genotypes [2]. Nevertheless, quantitative genetic considerations suggest that attention should be paid not only to a high mean of a potential breeding population, but also to a high variance and consequently response to selection. Both aspects are considered in the usefulness criterion (*UFC*, ref. [1]) which is defined as $UFC=µ+i\cdot h\cdot \sigma_{g}$, where µ denotes the mean of a breeding population, i denotes the intensity of selection to be applied in the breeding population, h denotes the square root of the heritability of a specific breeding population, and $\sigma_{g}$ denotes the genetic standard deviation of the breeding population resulting from a particular cross. It follows from the definition of the *UFC* that a cross with a low mean can still be promising because of a greater selection response. There are some pre-genomic studies that employed the *UFC* to empirically compare crossing strategies in maize breeding as an example [3,4]. Nevertheless, the crucial aspect for parental selection, the prediction of the usefulness of a cross, has remained an unsolved problem: While µ can be predicted with high confidence from the midparent value in an additive genetic model, predicting $\sigma_{g}$ for more complex traits has been challenging [5].

With the advent of low-cost genome-wide marker systems and the successful application of genome-wide prediction in plant breeding [6,7,8], the prediction of family means and variances based on marker effects, the components of *UFC*, returned to the forefront of quantitative genetic research. Zhong and Jannink [9], in a pioneering study based on computer simulations, investigated the potential and limitations of predicting means and variances of crosses based on marker effects. The prediction of family means was determined as the average predicted value of parents. Variance was predicted as the sum of the variances of the genetic effects across the segregating quantitative trait loci and twice the summed covariances between all pairs of segregating quantitative trait loci. For the prediction of variance, the recombination frequency of each pair of segregating quantitative trait loci was considered [9].

Subsequent studies have presented approaches to predict family means and variances by simulating progeny populations derived from specific crosses and determined the relevant population parameters by predicting the performances of each genotype belonging to the family based on marker effects [10]. Similar approaches for selecting parents based on their simulated progeny were proposed by Mohammadi et al. [11], Lado et al. [12], and Yao et al. [13]. Lehermeier et al. [14] derived family variances analytically as Bayesian estimates of genetic variances, assuming known allele substitution effects at all quantitative trait loci, and integrated this approach into the prediction of *UFC* [15]. The analytic approach of Zhong and Jannink [9] was implemented in a simulation study and a small-scale empirical experiment [16,17]. All the aforementioned studies showed very promising results but assumed that marker effects were known [10,11,12,14,15,16] or estimated marker effects considering genomic and phenotypic data of the populations whose *UFC* should be predicted [17]. In a study on cassava [18], marker effects and *UFC* were estimated in separate training and testing populations, yielding disappointing results.

Here, we present a validation study for the analytical approach of Zhong and Jannink [9] using four years of data from a barley breeding program. Our experiment aimed to evaluate the benefit and ability to predict the family mean, the family variance, and the *UFC* for selecting superior parents. The underlying data allowed us to design different validation scenarios that resemble situations on the basis of which breeders make decisions. The scenarios were implemented for three traits with different heritability and complexity.

## 2. Results

### 2.1. Quality of Phenotypic Data

**Figure 1:**
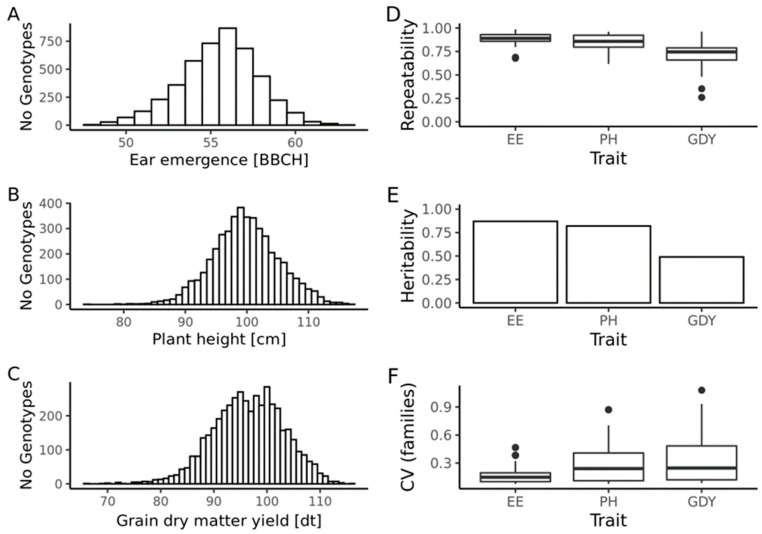
Distribution of phenotypic records (**A**–**C**), distribution of repeatabilities at test environments (**D**), heritabilities (**E**), and coefficients of variation (CV) of the families (**F**) for the traits ear emergence (EE), plant height (PH) and grain yield (GDY).

In total, 4488 genotypes were phenotyped in 38–40 environments, with the number of genotypes per environment varying from 72 to 1163. On average, every genotype was tested in 4.3, 3.5, and 3.2 environments for ear emergence, plant height, and grain yield, respectively. All traits approximated a normal distribution and the repeatability estimates ranged for ear emergence, plant height, and grain yield from 0.67 to 0.98, 0.61 to 0.96, and 0.18 to 0.96, respectively (Figure 1). The BLUEs across environments ranged for ear emergence from 47.8 BBCH [19] to 62.9 BBCH with a mean value of 55.7 BBCH, for plant height from 72.0 cm to 115.4 cm with a mean value of 98.0 cm, and for grain yield from 62. 6 dt ha^−1^ to 114.1 dt ha^−1^ with a mean value of 91.7 dt ha^−1^. Heritability estimates ranged from moderate (grain yield: $h^{2}=0.49$) to high (ear emergence $h^{2}=0.84$; plant height: $h^{2}=0.77$). The coefficient of variation for families was lowest for ear emergence, followed by plant height, and grain yield. Summarizing, for all three traits, the quality of the phenotypic data was excellent, providing a solid basis for our study.

### 2.2. Genome-Wide Prediction of Line Performances within and across Breeding Cycles

**Figure 2:**
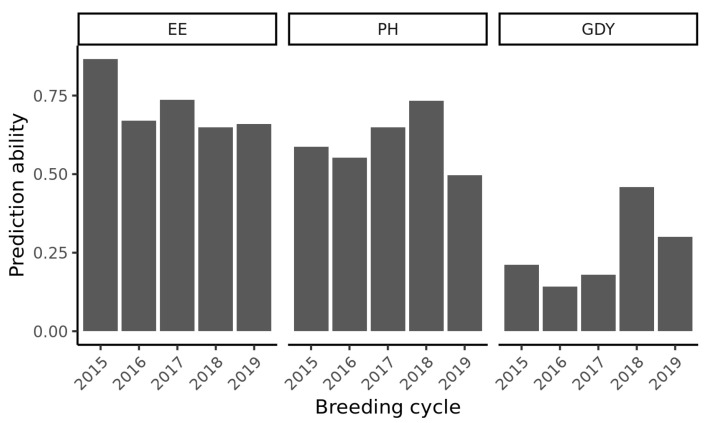
Prediction abilities for the traits ear emergence (EE), plant height (PH) and grain yield (GDY) across breeding cycles, where each breeding cycle was predicted based on a training set comprising only genotypes from the remaining breeding cycles.

Five-fold cross-validations were executed for each trait with the complete data set comprising all breeding cycles. Prediction abilities were 0.81 for ear emergence, 0.79 for plant height, and 0.73 for grain yield. Furthermore, leave-one-cycle-out cross validations were conducted and performances of the genotypes from one breeding cycle were predicted based on phenotypic data from genotypes of the remaining cycles (Figure 2). The prediction abilities for ear emergence ranged from 0.65 to 0.87, with the lowest prediction ability for the cycle of 2018 and the highest prediction ability breeding cycle of 2015. For plant height, the prediction abilities ranged from 0.55 to 0.73, with the minimum observed in the cycle of 2016 and the maximum prediction ability in the cycle of 2018. With a range from 0.14 to 0.46, the prediction abilities for grain yield were the lowest among the investigated traits. The highest prediction ability was observed for the cycle of 2018 and the lowest for the breeding cycle of 2016.

### 2.3. Prediction of the Family Mean, the Family Variance, and the Usefulness Criterion

**Figure 3:**
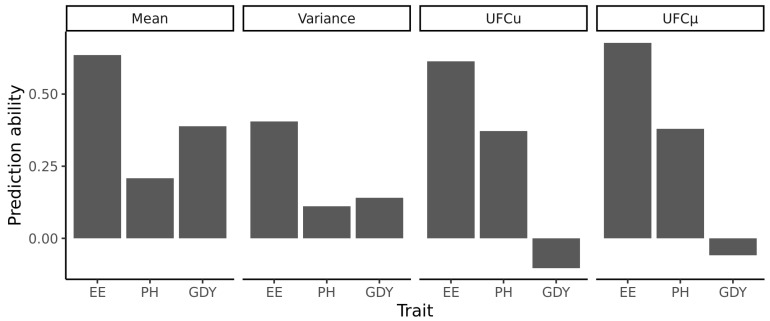
Prediction abilities for the population paramters family means, variances, usefulness criterion (*UFC*) predicted by *UFC* (*UFCu*) and *UFC* predicted by the mean (*UFCµ*) for the traits ear emergence (EE), plant height (PH) and grain yield (GDY) with the training set being identical to the test set employing the complete data set of the breeding program.

For the first scenario, the complete data set was used, and the training set was identical to the prediction set. Moderate to high correlations were observed between the predicted and the observed family means. The highest prediction ability was found for the trait ear emergence and the lowest for plant height (Figure 3). The prediction abilities for family means were 0.64 for ear emergence, 0.21 for plant height, and 0.39 for grain yield. In all cases, genomic predictions of the family means performed better than the prediction of the family means based on the midparent value derived from phenotypic records of the parents alone (no implementation of genomic prediction). For the latter, prediction abilities of 0.57, 0.18, and 0.17 were observed for ear emergence, plant height, and grain yield, respectively.

Low to moderate values were observed for the correlations between the predicted and the observed family variance. The correlation between the predicted and the observed variance was 0.41 for ear emergence, 0.11 for plant height, and 0.14 for grain yield (Figure 3). Correlations between the predicted and observed usefulness criterion ranged from −0.10 for grain yield to 0.61 for ear emergence (Figure 3).

In the second scenario, the final breeding cycle of 2019 was predicted based on a training set consisting of all previous breeding cycles (Appendix A Table A1). The prediction abilities for family mean ranged from 0.31 for grain yield to 0.64 for plant height. For family variance, the prediction abilities ranged from 0.12 for plant height to 0.44 for ear emergence. The prediction abilities for UFCu ranged from −0.15 for grain yield to 0.67 for ear emergence. For UFCµ, the prediction abilities ranged from −0.13 for grain yield to 0.68 for ear emergence.

**Figure 4:**
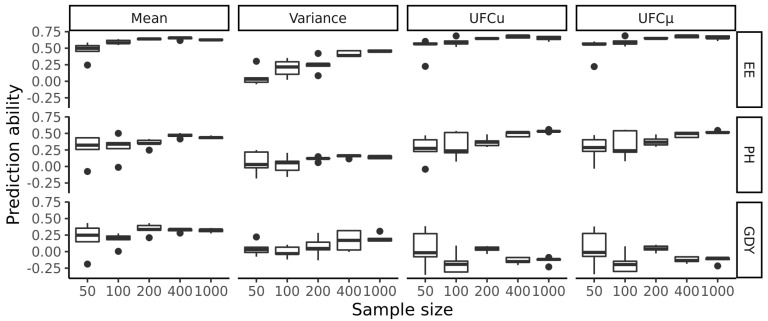
Prediction abilities for family means, variances, usefulness criterion (*UFC*) predicted by *UFC* (*UFCu*) and *UFC* predicted by mean (*UFCµ*) for the traits ear emergence (EE), plant height (PH), and grain yield (GDY). The population parameters of the last breeding cycle were predicted based on data from the previous breeding cycles. Training populations of sizes ranging from 50 to 1000 were randomly sampled five times.

To investigate the influence of population size on the prediction ability of family means, family variances and the usefulness criterion (*UFC*), a leave-one-cycle-out validation was performed for the final breeding cycle of 2019 using randomly sampled training sets from the previous breeding cycles with population sizes ranging from 50 to 1000 genotypes (Figure 4). For ear emergence, prediction abilities improved with increased training population size for means, variances, and the *UFC*. We observed that the prediction abilities improved steadily for population sizes ranging from 50 to 200 genotypes and then stagnated at a median value of approximately 0.67. Towards the maximum population size, the variation in prediction abilities was smaller. For the prediction of the genetic variance, a clear upwards trend in prediction abilities was observed at higher sizes of the training set. Nevertheless, no clear trend was observed in the variations of prediction abilities. Similar trends were observed for both ways of predicting *UFC*, either using predicted family means only or using predicted family means and variances: Prediction abilities increased from a training set population size of 50 to 200, where they settle at a stable level. As the size of the training population increased, the variation of prediction abilities decreased.

For plant height, the trends for the prediction of means and both ways of predicting the *UFC* were similar (Figure 4). A decreasing distribution of prediction abilities was observed for the prediction of genetic variances, but absence of clear upwards trend of prediction abilities with higher training set population sizes.

We observed a different situation for grain yield. While the trends for predicting family means were similar to the findings for ear emergence and plant height, the trends for the other measures to be predicted drew a more complex picture. As the size of the test populations increased, the prediction abilities for genetic variances improved slightly. In contrast, no clear trend was observed for the variation of prediction abilities. Both methods for predicting the *UFC* showed a decrease in the variation of the prediction abilities with increasing size of the training populations (Figure 4). The median values showed neither an upward nor a downward trend and moved towards zero as the training population increased.

## 3. Discussion

Besides the mean, the genetic variance of the family resulting from a cross is the most important factor for its superiority and is therefore considered in the *UFC*. Despite an increasing number of publications proposing to predict the family mean and variance for a specific cross based on marker effects, proof of concepts based on comprehensive data sets are scarce. A recent attempt used a dataset from a cassava breeding program and yielded disappointing results [18]. In our study, we investigated the potential and limitations of predicting the *UFC* to identify optimal parent combinations using a data set generated in the course of commercial winter barley breeding. Despite the extensive population size, the use of data from commercial breeding programs also has some drawbacks. For a sufficiently precise study of the genetic variance of families, large and equal family sizes are ideal. The population composition in the present study has not been designed for scientific purposes, so family sizes vary. The contribution of parents to families also varies, and phenotypic selections at early stages can bias estimates of population parameters. The imbalanced nature of the data set made it impossible to consider family-specific genotype-times-environment effects and heterogeneous residual variances which could as well be a source of bias in the estimation of within family variances. On the other hand, the extensive data allow initial validation of the potential of predicting the *UFC*.

### 3.1. Leave-One-Cycle-Out Cross-Validations Revealed That Performances of Individual Genotypes Can Be Predicted across Breeding Cycles

Five-fold cross-validations showed high prediction abilities for individual genotype performances for all three traits and amounted to 0.81 for ear emergence, 0.79 for plant height, and 0.73 for grain yield (Figure 2), which was consistent with similar findings from experiments in an elite winter barley panel [20]. For leave-one-cycle-out validation, prediction abilities were lower for most breeding cycles for all three traits, averaging 89% for ear emergence, 75% for plant height, and for grain yield 36% of the five-fold cross-validation scenario that included all breeding cycles combined. For most traits, within cycle genome-wide prediction abilities were reported to be higher than across cycle genome-wide prediction abilities in sugar beet (*Beta vulgaris* L.) [21], maize (*Zea mays* L.), wheat (*Triticum aestivum* L.) [22], and in rye (*Secale cereale* L.) [23]. Except for the breeding cycle of 2015 including a relatively large number of frequently used parental genotypes, which exceeded the prediction ability of the five-fold-cross-validation for ear emergence, no breeding cycle showed higher prediction abilities compared to the five-fold cross-validation. The relatively low prediction abilities for grain yield may be attributed to the comparably low heritability. Additionally, it can be hypothesized that genotype-by-environment interactions play a larger role in this trait, which was shown in a similar data set for barley [24]. Summarizing, the findings indicated that genomic prediction of the performance of individual genotypes is working well for the underlying data set.

### 3.2. Prediction Abilities of the Family Mean across Cycles Were Lower than Reported in Previous Simulation Studies

In an additive model, it is expected that the mean performance of the parents, i.e., the midparent value, is equal to the mean of the progeny of the respective cross [25]. In our study, the mean of a family was predicted based on the midparent value of genomic estimated breeding values (GEBVs). The correlations were substantially lower than the values reported from the literature. In previous studies, the family means have been predicted based on midparent values based on GEBVs of parental lines for simulated progeny populations [10,11]. In these studies, correlations between the predicted family mean and the family mean of the simulated progeny population was 0.95 for silking date and 0.91 for protein content in *Zea mays* [10], or 0.89 for deoxynivalenol and grain yield in barley [11]. One drawback of these studies is the fact that the simulated progeny populations which functioned as test set were generated based on the same prediction models as the midparent GEBVs that were designed to predict the family mean. Clearly, this concept does not reflect a situation a breeder faces before deciding which crosses to produce. Osthushenrich et al. (2018) observed a correlation of 0.95 for grain yield in a 5×5 factorial design tested in an augmented field design at 5 locations in one year, where the training set was identical to the test set. Neyhart and Smith [26] reported moderate prediction abilities in barley, amounting to 0.46 for *Fusarium* head blight, 0.62 for heading date, and 0.53 for plant height. High prediction abilities of the family mean have been reported in maize with 0.91 for plant height, 0.83 for ear height, and 0.80 for silking date [27]. In the present study, the prediction ability of the mean was lower for all traits, even if the training set was identical to the test set (Figure 3). This can be explained by varying sizes of families, an unbalanced design of field tests, where parents have usually not been tested in the same environments as the progeny populations, and in the case of grain yield by a relatively low heritability.

In another scenario, mimicking the typical situation in breeding, the training set consisted of genotypes from previous cycles and the test set consisted of genotypes from the last cycle of the breeding program. For plant height, the prediction ability was higher compared to the above-described scenario with the training set being identical to the test set (Table A1). For ear emergence and grain yield, prediction abilities were comparable. To examine the influence of the training set population size, 50, 100, 200, 400, and 1000 genotypes were sampled from the previous cycles of 2016, 2017, and 2018 to predict the final cycle of 2019. At the maximum training population size of 1000 genotypes, the mean prediction abilities for the family mean were 0.62 for ear emergence, 0.43 for plant height, and 0.33 for grain yield (Figure 4). For larger sizes of training populations, the median values of the prediction abilities approach $\surd h^{2}$, which corresponds to the theoretical limit of prediction abilities [28].

### 3.3. Low Ability to Predict the Genetic Variance of Families

The prediction abilities for the genetic variance of families were lower compared to the prediction ability for the family mean for all traits (Figure 3). This trend is consistent with previous results from experiments predicting the genetic variance based on simulated populations. In maize, the prediction abilities for plant height, ear height, and silking date amounted to 0.03, −0.24, and 0.14 [27], and 0.01, 0.39, and 0.48 for *Fusarium* head blight severity, heading date, and plant height, respectively [26]. In both studies, progeny populations of potential crosses were simulated based on estimated marker effects obtained from a training population, and validations were examined through phenotypic analysis of families in subsequent field trials. The results presented in this study were used to validate the prediction ability of family variance based on the analytic approach proposed by [16]. In a field trial-based validation of the analytical approach, this method led to prediction abilities of 0.34 or 0.76 if downwards outliers were removed [17]. However, this scenario does not reflect the scenario breeders typically face, as the training set and the test set were identical.

### 3.4. Prediction of the Usefulness Criterion

Depending on the trait, the trends for the prediction abilities of the *UFCu* varied strongly. While the prediction abilities for the family variance were lower than those for the family mean in all cases, the prediction abilities for the *UFCu* were comparable to those observed for family mean in ear emergence, higher for plant height, and even negative and therefore lower for grain yield. Since both the mean and the variance determine the value of the *UFC*, it is reasonable to assess the correlation of both measures. For all investigated traits, the correlations between mean and variance were negative for the observed (−0.28 for ear emergence, −0.26 for plant height, and −0.72 for grain yield), as well as for the predicted values (−0.29 for ear emergence, −0.15 for plant height, −0.47 for grain yield), and grain yield showed the lowest correlations in both cases. Several studies report a triangular relationship of mean and genetic variance, where families with extreme, i.e., very low and very high means are associated with low genetic variance, and families with intermediate means are associated with high genetic variance [11,26]. This phenomenon was not observed as clearly in the present study.

The coefficients of variation, i.e., the ratio of the standard deviation to the mean, of the families for ear emergence was comparably low in the present breeding program (Figure 1F). It can be presumed, that the influence of the family variance on the predicted and the observed *UFC* was relatively low. This explains the small difference between the prediction abilities for family mean and *UFCu*.

For plant height, the coefficients of variation of the families were higher compared to the values observed for ear emergence. Consequently, for plant height, the contribution of the family variance to the *UFC* should be larger compared to ear emergence. Even though the prediction ability for family variance was lowest for plant height, the prediction ability of the *UFCu* is larger compared to the prediction ability of the family mean. In comparison to ear emergence and grain yield, the correlations between variance and mean were highest for plant height for observed, as well as for predicted values. Together with the relatively high heritability of the trait of $h^{2}=0.82$, these findings explain the relatively high prediction ability observed for the *UFCu*.

The highest coefficient of variation of the families were observed for grain yield. Similarly, to plant height, this finding points to a relatively large impact of the family variance on the *UFC*. Considering the notable low correlation between predicted family mean and predicted family variance, it is not surprising to observe a major difference in prediction ability of the *UFCu* compared to the prediction of the family mean. Furthermore, in contrast to both previously discussed traits, the phenotypic records of grain yield disclosed a relatively low heritability of $h^{2}=0.49$. The high complexity of the trait grain yield, as well as the resulting quality of phenotypic data, lead to higher degrees of uncertainty in the estimation of marker effects, which are known to play a larger role in second degree statistics, e.g., family variance, as compared to first degree statistics which include the family mean.

Because of the uncertainty in variance prediction discussed above and reported in the literature [18], and the fact that the family mean generally contributes more to the *UFC* than the standard deviation, which is multiplied by the square root of heritability and the selection intensity, the *UFC* was furthermore predicted based on the family mean only, i.e., *UFCµ*. In general, the prediction abilities of the *UFCu* and the *UFCµ* deviated only slightly. For ear emergence and grain yield, prediction abilities increased while it was comparable for plant height (+10% for ear emergence, +3% for plant height, and +13% for grain yield). The relatively low differences between both approaches of predicting the *UFC* are due to the comparably low influence of the family variance on the *UFC* under the given conditions of the breeding program.

**Figure 5:**
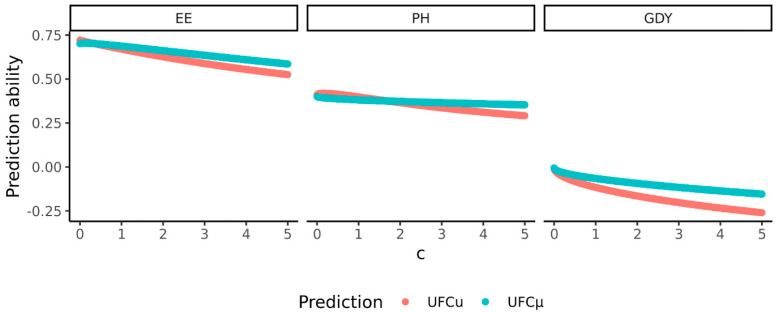
Prediction ability for the *UFC* varies depending on the complexity of the trait (ear emergence (EE), plant height (PH), and grain yield (GDY)), and the product of the square root of heritability and selection intensity given as the constant *c*. Two methods of predicting the *UFC* were applied: *UFCu* (red) and *UFCµ* (blue).

To further elucidate the impact of the selection intensity and the square root of heritability on the prediction abilities of both approaches of predicting the *UFC*, the product of both constants was assumed as c=i·h, with levels ranging from 0 to 5. For example, at a heritability of $h^{2}=1.0$, c will be 0 if no selection is applied, 1 at a selection fraction of 38%, 2.5 at a selection fraction of 1.61%, and 5 at a selection fraction of 1 out of 1000,000. The constant *c* was used to calculate the observed *UFC*. Correlations between these hypothetical values of the *UFC* and the predicted values indicate that for all three traits, under medium to high selection intensities, prediction abilities will be higher for the *UFCµ* than the *UFCu*. Both approaches performed similarly for ear emergence and grain yield at very low selection intensities and for plant height at low to medium selection intensities (Figure 5). If at all, a small benefit of the *UFCu* is only given for ear emergence and plant height under the circumstance of low selection intensities. For grain yield, no benefit of predicting the *UFCu* was observed under any selection intensity.

In a breeding program, predictions are based on the inference resting on genomic and phenotypic data from previous cycles or genetically independent populations. Therefore, a further scenario was considered in the present study, in which the performances of the final breeding cycle were predicted based on the information on the genotypes originating from all previous cycles. In this scenario, *UFCu* performed slightly better than *UFCµ* for plant height. For the remaining traits, trends were similar to the scenario based on the full data set with the prediction and test set being identical. Moreover, to elucidate the influence of the population size, subsamples of the population sizes 50, 100, 200, 400, and 1000 genotypes were drawn which were then used as training set to calibrate models for predicting the final breeding cycle. These experiments confirmed the previously discussed findings of the present study, based on the full data set with the prediction and test sets being identical. In general, larger training set population sizes led to decreased dispersion of prediction abilities for all traits. For nearly all predicted population parameters, increasing population size leads to higher prediction abilities except for grain yield, where both approaches of predicting the *UFC* remain around zero for all training set population sizes. In all cases, the difference between the prediction abilities of the *UFCu* and the usefulness criterium predicted by the family mean was low.

The results presented in the underlying study largely correspond to findings obtained from a cassava breeding program [18]. In cassava, median prediction abilities for the usefulness criterion predicted by the *UFC* ranged from 0.1 to 0.83 in a cross-validated scenario and 95% of the prediction abilities were greater than zero, assuming a heritability of $h^{2}=1$ and varying selection intensities per family. Wolfe et al. [18] similarly described low differences between the prediction abilities for the family mean and the *UFC* and reported a high correlation of both.

## 4. Materials and Methods

### 4.1. Plant Material and Field Trials

The plant material used in this study is based on the winter barley breeding program of KWS LOCHOW GmbH (Bergen, Germany) and comprises in total 4500 winter barley lines. Each genotype was generated based on a double-haploid technology using two-, three-, and four-way crosses. Double-haploids were generated using F_1_ plants. Genotypes, which originate from the same cross were denoted as a family. In total, a number of 347 families were part of the barley breeding program. The underlying data comprise four breeding cycles, corresponding to the year in which they were phenotyped for the first time. Barley breeding in Europe is not based on closed second breeding within companies. The use of lines also from other breeding programs in combination with the different time for doubled haploid production due to an additional generation for 3- and 4-way crosses compared to 2-way crosses leads to a complex pedigree structure and the parents of the cycles do not necessarily follow each other linearly.

Phenotypic evaluation took place in the years 2015, 2016, 2017, 2018, and 2019 for the traits grain yield (dt ha^−1^), plant height (cm), and ear emergence (BBCH; [19]) in up to 10 locations. The experimental design of the field trials followed alpha lattice designs. A subset of 433, 1026, 1021, 1020, and 1000 lines were tested in the year 2015, 2016, 2017, 2018, and 2019 in two to four replications. Only sparse information was available for the parents of the first breeding cycle (2015). The dataset was nevertheless considered because it contained information on genotypes that were frequently used as parents in subsequent cycles.

### 4.2. Genomic Data

An Illumina Infinium 5 k SNP array was used to genotype the lines [3,29]. The mean rate of missing values was 1.4%. Markers with a minor allele frequency of 0.05 or less were excluded. After quality control, SNP markers with a missing rate lower than 5% were imputed based on the allele frequency. The original data set comprised 4501 markers from which 2898 remained after quality control and were used for further analysis.

### 4.3. Phenotypic Data Analysis

For the analysis of the phenotypic data, we implemented a two-stage approach. After removing outliers following the method of Tukey and Anscombe [30], a linear mixed model was used to analyze the data for each environment:

(1) $$y=1_{n}µ+Zg+Z_{B}b+Z_{R}r+e,\;$$

where *y* denoted the vector of phenotypic values for each genotype tested in the specific environment, 1*_n_* denoted the *n*-dimensional vector of 1’s and *n* denoted the number of records in the specific environment, *µ* was the common intercept, *g* denoted the vector of genotypic values of the lines tested in the specific environment and was considered as random effect, *r* denoted the vector of replication effects, considered to be random, and *b* was the incomplete block effect, which was considered as random effect, and *e* denoted the random residual. *Z*, $Z_{B},$ and $Z_{R}$ were design matrices for *g*, *b*, and *r*, respectively. We assumed that all random effects followed an independent normal distribution with different variance components for genotype, replication, and block effects. Repeatability was estimated for each environment as:

(2) $$repeatability=\frac{\sigma_{g}^{2}}{\sigma_{g}^{2}+\frac{\sigma_{e}^{2}}{n_{R}}},$$

where $\sigma_{g}^{2}$ denoted the genotypic variance, $\sigma_{e}^{2}$ denoted the residual variance, and $n_{R}$ denoted the average number of replications per genotype. The best linear unbiased estimations (BLUEs) for genotypes within each environment were obtained using model (1) assuming fixed genotypic effects.

The BLUEs of the genotypes in each environment were used in a second step to fit a further linear mixed model across the environments:

(3) $$y=1_{m}µ+Zg+Z_{E}u+e,\;$$

where y denotes the vector of BLUEs that were calculated in the first step for the genotypes in each environment. $1_{m}\;$ denotes a vector of 1’s with the length of *m* which refers to the total number of genotypes across all environments, *µ* denotes the common intercept, *g* denotes the vector of genotypic effects for all genotypes, *u* denotes the vector of environmental effects, and *e* denoted the vector of residuals. *Z* and $Z_{E}$ denote corresponding design matrices for *g* and *u*, respectively. *µ* was assumed to be a fixed parameter, while *g*, *u*, and *e* were assumed to follow an independent normal distribution. The resulting estimated variance components were used to calculate the broad-sense heritability as:

(4) $$h^{2}=\frac{\sigma_{G}^{2}}{\sigma_{G}^{2}+\frac{\sigma_{e}^{2}}{\;n_{E}}},\;$$

where $n_{E}$ denotes the average number of environments in which the genotypes were evaluated. Furthermore, the genotypic effects were assumed to be fixed in model (3) in order to calculate the BLUEs across environments.

The genetic variance of the families that were tested in the field was obtained by the following model:

(5) $$y=1_{n}µ+Zg+Z_{E}u+Z_{EB}b+Z_{ER}r+e,$$

The genotypic variances were estimated separately for each family by assuming $g\sim N\left(0,{⊕}_{k=1}^{f}G_{k}\right),\;G_{k}=I_{k}\sigma_{G_{k}}^{2}$ nd $\sigma_{G_{k}}^{2}$ was the genotypic variance for *k*-*th* family. $Z_{EB}$ and $Z_{ER}$ were design matrices for block and replication effects nested into environments, respectively. A model considering family specific variance for genotype-times-environment interaction effects and heterogeneous residual variance was attempted in the first place but did not converge. For all mixed linear models that were applied in the phenotypic analysis, ASReml-R [31] was employed.

The *UFC* was estimated for each family as the sum of the family mean and response to selection [1]. Response of selection was estimated assuming a selection intensity of i=1.27 and a fixed broad-sense heritability for all families observed in the phenotypic data analyses, because the non-orthogonal data set led to convergence problems and prevented the family-specific estimation of heritability.

### 4.4. Genome-Wide Prediction within and across Breeding Cycles

The ability of genomic prediction was evaluated using genomic best linear unbiased prediction (GBLUP, [32]). The GBLUP model was given by $y=1_{n}µ+g+e$, where *y* denoted the vector of BLUEs of the parental genotypes, $1_{n}$ denoted an *n*-dimensional vector of 1’s, *n* was the number of genotypes, *µ* denoted the common intercept, g denoted the vector of genotypic values, *e* denoted the residual term. We assumed that $g\sim N\left(0,G\sigma_{g}^{2}\right)$, where *G* denoted the *n*-dimensional genomic relationship matrix [32] and $e\sim N\left(0,I\sigma_{e}^{2}\right)$.

To assess the prediction abilities within the breeding program under study, five-fold cross validations were performed for the entire data set across all breeding cycles. For this purpose, the lines of the breeding program were randomly divided into five subsets, four of which were used as training sets and the fifth as prediction set. The prediction ability was examined as the correlation between BLUEs and predicted genotypic values for the test set. This procedure was repeated 100 times and the mean prediction ability was obtained as the final prediction ability, $r_{GP}$.

The prediction ability between breeding cycles was investigated by dividing the full data set into the single breeding cycles, i.e., 2015, 2016, 2017, 2018, and 2019. Subsequently, the data of four breeding cycles were used as the training set to predict the genotypic values of the remaining breeding cycle, which functioned as the test set. This procedure was repeated for all breeding cycles.

To estimate the additive effects of single markers, an RRBLUP model with the form $y=1_{n}µ+X\alpha +e$ was applied, where α was the vector of additive effects of markers assuming $\alpha \sim N\left(0,I_{p}\sigma_{\alpha }^{2}\right)$, $e\sim N\left(0,I_{n}\sigma_{e}^{2}\right)$. $I_{p}$ and $I_{n}$ were identity matrices of order p and n, with p being the number of markers and n being the number of genotypes.

### 4.5. Prediction of the Family Mean

The genomic estimated breeding values (GEBV) of each genotype were obtained by the above-mentioned genomic prediction models, GBLUP. To predict the mean of a progeny of a cross, the mean between the genotypes employed as parents was calculated, where the parents were weighted for the expected proportion of contributed genome. For comparison, the midparent value calculated from the phenotypic records of the parents alone was used as a point of reference.

### 4.6. Prediction of the Family Variance

For the prediction of the family variance, the method suggested by [16] was applied. Briefly, marker effects were estimated using the above-mentioned genomic prediction model, RRBLUP. The predicted variance was then obtained from the estimated marker effects by the following formula:

(6) $$\sigma_{G}^{2}=var\left(S\right)=\sum_{c}\sum_{j,k}cov\left(S_{j},S_{k}\right),\;$$

where S denotes the lines of a family, $S_{j}$ is two times the additive effect of the maternal or paternal allele, c is summed over the number of chromosomes, j is summed over the number of loci of a chromosome, and j,k is the sum of all locus pairs of a chromosome. The covariance of two linked loci was given by

(7) $$cov\left(S_{j},S_{k}\right)=\left(\frac{1}{2}q_{jk}-\frac{1}{4}\right)\left(m_{j}m_{k}+v_{j}v_{k}-m_{j}v_{k}-v_{j}m_{k}\right),\;$$

where $m_{j}$ and $m_{k}$ denote the effect of maternal alleles at the loci j and k, respectively and $v_{j}$ and $v_{k}$ denoted the effect of paternal alleles at the loci j and k, respectively. Following Equation (20) of Osthushenrich et al. [16], the parameter $q_{jk}$ is a function of the linkage disequilibrium between two loci and was calculated for each pair of linked loci assuming zero generations of random mating.

### 4.7. Prediction of the Usefulness Criterion

The *UFC* was predicted for each family as the sum of the predicted family mean and the predicted response to selection [1]. The response to selection was predicted using the predicted family variance, and assuming a selection intensity of i = 1.27 as well as a fixed broad-sense heritability for all families as outlined above. For predicting the *UFC*, square root of heritability was assumed to be 1. To assess the prediction ability, the correlation of the predicted and the observed *UFC* was calculated. This method of predicting the *UFC* is referred to as *UFCu*. As a further point of reference, *UFC* was additionally predicted using the predicted family means only, hereafter referred to as *UFCµ*.

### 4.8. Validating Predictions of the Family Mean and the Family Variance

In the first step, a scenario similar to the study design of [17] was investigated. Marker effects were estimated based on the full data set including all parents and families with available genotypic and phenotypic information. Predictions were validated with data from families derived from two-way crosses, assuming a minimum family size threshold of at least 10 genotypes and an estimated genetic variance greater than 0.01. Data were available from 66 families for ear emergence, 57 families for plant height, and 65 families for grain yield. The correlation between the observed mean and variance and the predicted mean and variance for the families was calculated.

Subsequently, methods for predicting the family means and variances were tested employing a leave-one-cycle-out validation for the final breeding cycle of 2019. The marker effects were estimated based on the full data set, excluding the data for the genotypes originating from the breeding cycle of 2019. The correlations between the observed mean and the observed variance of the family and the predicted mean and variance were calculated for the breeding cycle of 2019.

To elucidate the impact of the population size, an additional leave-one-cycle-out validation was executed. This time, the variances and means of the families from the final cycle of 2019 were predicted based on phenotypic data from the remaining cycles 2015, 2016, 2017, and 2018 with randomly sampled training sets comprising 50, 100, 200, 400, and 1000 randomly sampled genotypes.

## 5. Conclusions

The reliable prediction of the family variance and the *UFC* based on marker effects remain the pinnacle of any breeding intention. While the prediction of the family mean leads to acceptable or satisfying prediction abilities for all traits, the prediction of the family variance seems to be impeded by several uncertainties. No benefits were obtained for predicting the *UFC* based on analytic approaches, and in complex traits with low heritability, predictions might even harmfully lead to the wrong direction. Our data suggest that selections based on the *UFC* are not advisable in such cases. In accordance with existing literature on the prediction of family variance and the *UFC* based on realistic breeding scenarios, it can be concluded that the applied analytical methods are not well enough developed to be trustworthily recommended to breeders or decisionmakers in the breeding industry.

## Data Availability

The data can be provided by KWS LOCHOW GmbH pending scientific review and a completed material transfer agreement. Requests for the data should be submitted to Neele Wendler (neele.wendler@kws.com).

## Appendix A

**Table A1:** Prediction abilities of population parameters in a scenario where the test set corresponds to the final breeding cycle of 2019 and the training set corresponds to a combination of all previous cycles.

Trait	Mean	Variance	UFCu	UFCµ	N° Families
Ear emergence	0.64	0.44	0.67	0.68	21
Plant height	0.41	0.12	0.55	0.53	17
Grain yield	0.31	0.33	−0.15	−0.13	21
